# Different feeding patterns affect meat quality of Tibetan pigs associated with intestinal microbiota alterations

**DOI:** 10.3389/fmicb.2022.1076123

**Published:** 2022-12-02

**Authors:** Yanbin Zhu, Cidan-yangji, Guangming Sun, Chengzeng Luo, Jiujun Duan, Bin Shi, Teng Ma, Shanlong Tang, Ruqing Zhong, Liang Chen, Basang-wangdui, Hongfu Zhang

**Affiliations:** ^1^Institute of Animal Husbandry and Veterinary Medicine, Tibet Academy of Agriculture and Animal Husbandry Sciences, Lhasa, China; ^2^The State Key Laboratory of Animal Nutrition, Institute of Animal Sciences, Chinese Academy of Agricultural Sciences, Beijing, China; ^3^College of Animal Science, Xinjiang Agricultural University, Urumqi, China

**Keywords:** Tibetan pigs, meat quality, intestinal microbiota, feeding patterns, long-chain fatty acid

## Abstract

This study aimed to investigate the effects of different feeding patterns on meat quality, gut microbiota and its metabolites of Tibetan pigs. Tibetan pigs with similar body weight were fed the high energy diets (**HEP**, 20 pigs) and the regular diets (**RFP**, 20 pigs), and free-ranging Tibetan pigs (**FRP**, 20 pigs) were selected as the reference. After 6 weeks of experiment, meat quality indexes of semitendinosus muscle (**SM**) and cecal microbiota were measured. The results of meat quality demonstrated that the shear force of pig SM in FRP group was higher than that in HEP and RFP groups (*p* < 0.001); the pH-value of SM in HEP pigs was higher at 45 min (*p* < 0.05) and lower at 24 h (*p* < 0.01) after slaughter than that in FRP and RFP groups; the SM lightness (L* value) of FRP pigs increased compared with RFP and HEP groups (*p* < 0.001), while the SM redness (a* value) of FRP pigs was higher than that of RFP group (*p* < 0.05). The free fatty acid (**FA**) profile exhibited that the total FAs and unsaturated FAs of pig SM in HEP and RFP groups were higher than those in FRP group (*p* < 0.05); the RFP pigs had more reasonable FA composition with higher n-3 polyunsaturated FAs (**PUFAs**) and lower n-6/n-3 PUFA ratio than HEP pigs (*p* < 0.05). Based on that, we observed that Tibetan pigs fed high energy diets (HEP) had lower microbial α-diversity in cecum (*p* < 0.05), and distinct feeding patterns exhibited a different microbial cluster. Simultaneously, the short-chain FA levels in cecum of FRP and RFP pigs were higher compared with HEP pigs (*p* < 0.05). A total of 11 genera related to muscle lipid metabolism or meat quality, including *Alistipes*, *Anaerovibrio*, *Acetitomaculun*, etc., were identified under different feeding patterns (*p* < 0.05). Spearman correlation analysis demonstrated that alterations of free FAs in SM were affected by the genera *Prevotellaceae_NK3B31_group*, *Prevotellaceae UCG-003* and *Christensenellaceae_R-7_group* (*p* < 0.05). Taken together, distinct feeding patterns affected meat quality of Tibetan pigs related to gut microbiota alterations.

## Introduction

Tibetan pig is a rare plateau pig species, mostly living in the alpine and cold areas of semi-farming and semi-grazing (Yang et al., 2021). In addition to its developed cardiopulmonary function, strong lipid-settling ability, and ability to endure plateau anoxic environments, it has a high level of cold or stress resistance, delicious meat, and has a lot of fat between the muscles (Fan et al., 2016; Luo et al., 2022; Niu et al., 2022). In recent years, with the increasing demand for ecological, green and high-quality pork products, Tibetan pig breeding industry has attracted widespread attention.

The traditional way of feeding Tibetan pigs is grazing, which mainly relies on grass, fruits, roots and other food sources of grassland and understory, and rarely supplements with refined feed, so the breeding cost is low (Wang et al., 2013). Understory resources are abundant in summer and autumn, and a lot of nutrients can be obtained by Tibetan pigs; however, in winter and spring with cold climate, the food resources are gradually reduced, and the nutrients that can be absorbed are limited, far from meeting the growth needs of pigs (Zhang et al., 2019; Yang et al., 2021). This traditional feeding pattern seriously slows down the growth and development of pigs and reduces the production performance of Tibetan pigs. Changing the traditional breeding way, therefore, adopting the appropriate house feeding pattern is the only way for the development of industrialization of Tibetan pigs. However, whether changing the way of Tibetan pig breeding will change the quality of meat products needs further verification, especially the effects of diet transformation on Tibetan pork quality from extensive farming to fine farming needing to be clarified.

In recent years, with the depth exploration of microbial functions, gut-brain axis, gut-liver axis and other signaling pathways mediated by microorganisms and their metabolites (especially short-chain fatty acids) have been confirmed to be involved in the host’s energy metabolism (Morais et al., 2021; Wang et al., 2021). The microbiota-gut-skeletal muscle axis has also been gradually confirmed, and dietary components (including dietary fiber) can change the gut microbiota to regulate the glucose and lipid metabolism of the host skeletal muscle, resulting in the improvement of their meat quality (Sonnenburg and Backhed, 2016; Chen et al., 2022). Therefore, it is a new idea to regulate skeletal muscle metabolism by altering intestinal microbiota or its metabolites through diet and other factors, ultimately improving muscle production and quality.

Therefore, this study aimed to explore the effects of different feeding patterns on meat quality of Tibetan pigs, and analyze the role of cecal microorganisms and their metabolites short-chain fatty acids (**SCFAs**), which is expected to provide a theoretical basis for large-scale breeding of Tibetan pigs, and provide a reference for selecting specific feeding patterns according to different market orientation and demand structure to achieve accurate pig breeding.

## Materials and methods

### Ethics approval

The experimental protocol (Ethics Approval Code: IAS2021-241) was reviewed and approved by the Institutional Animal Care and Use Committee of the Institute of Animal Sciences, Chinese Academy of Agricultural Sciences (Beijing, China).

### Animals, experimental design, and sample collection

This experiment was conducted in the research farm (Shannan District, Tibet, China) of Institute of Animal Husbandry and Veterinary Medicine, Tibet Academy of Agriculture and Animal Husbandry Sciences. A total of 40 house-feeding Tibetan pigs [initial body weight (**IBW**) = 22.4 ± 1.7 kg] with the same genetic background were allotted to 2 pens randomly and each pen was regarded as one dietary treatment. During the 42-day experimental period, Tibetan pigs were fed the regular Tibetan pig diets (**RFP** group) and the high energy content diets (**HEP** group; Table 1). All pigs were allowed to free access to feed and water. Diets were formulated to meet or exceed the vitamins and minerals of pigs according to the nutrient requirements of swine (GB/T 39235–2020). At the same time, another 20 free-ranging Tibetan pigs (IBW = 21.3 ± 1.0 kg) were selected and set as the reference (**FRP** group).

**Table 1:** Composition and nutrient level of experimental diet (as fed-basis).

Ingredients	RFP diet	HEP diet
Corn	32.14	66.4
Soybean meal	7.5	15.5
Wheat bran	7.26	15
Alfalfa	50	0
Limestone	1	1
CaHPO4	0.5	0.5
NaCl	0.3	0.3
L-Lys (70%)	0.2	0.2
Choline chloride	0.1	0.1
Premix^1^	1	1
Total	100	100
**Calculated nutrient levels %**		
CP	15.37	14.83
DE, MJ/kg	9.77	13.9
SID Thr	0.43	0.43
SID Trp	0.12	0.13
SID Lys	0.64	0.75
SID Met	0.2	0.23
Ca	1.09	0.56
TP	0.42	0.46
STTD P	0.25	0.25

1 Provided the following quantities per kg of diet: vitamin A, 9140 IU; vitamin D_3_, 4,405 IU; vitamin E, 11 IU; menadione sodium bisulfite, 7.30 mg; riboflavin, 9.15 mg; D-pantothenic acid, 18.33 mg; niacin, 73.50 mg; choline chloride, 1,285 mg; vitamin B_12_, 200 μg; biotin, 900 μg; thiamine mononitrate, 3.67 mg; folic acid, 1,650 μg; pyridoxine hydrochloride, 5.50 mg; I, 1.85 mg; Mn, 110.10 mg; Cu, 7.40 mg; Fe, 73.50 mg; Zn, 73.50 mg; Se, 500 μg.

RFP: feeding the regular Tibetan pig diets; HEP: feeding the high energy content diets. CP: crude protein; DE: digestible energy; SID AA: standardized ileal digestible amino acid; TP: total phosphorus; STTD P: standardized total tract digestibility of phosphorus.

At the end of this experiment (day 42), 7 randomly selected pigs of each treatment were sacrificed by electric stunning and carcass weight (**CW**) of each pig was recorded. An incubation period of 3 h at room temperature was followed by centrifugation at 3,000 rpm for 10 min after drawing blood from the jugular vein with a sterilized syringe. We aliquoted and stored the serum samples at −80°C for subsequent analysis. The semitendinosus muscle (**SM**) samples (approximately 1 g) were collected into 2-mL sterile tubes for free fatty acids analysis. Aseptically collected chyme from the cecum was sequenced for microbial 16S rRNA genes and analyzed for SCFA levels. All samples of SM and cecum digesta were immediately frozen in liquid nitrogen and stored at −80°C.

### Analysis of serum metabolites

Concentrations of low-density lipoprotein cholesterol (**LDL-C**, Cat # A113-2-1), alanine aminotransferase (**ALT**, Cat # C009-3-1), alkaline phosphatase (**ALP**, Cat # A112-1-1) and high-density lipoprotein cholesterol (**HDL-C**, Cat # A086-1-1) in serum were measured by commercial assay kits from Nanjing Jiancheng Bioengineering Institute (Nanjing, China).

### Measurement of meat quality index

Meat color and pH value of SM samples were directly measured by OPTO-STAR and pH-STAR (Matthäus, Germany) according to the manufacturer’s instructions at 45 min and 24 h postmortem, respectively. The shear force value of SM samples was obtained by the Warner-Bratzler meat shear machine (Salter 235, Manhattan, Kansas, United States) following the procedure described by Lang et al. (2020).

### Detection of free fatty acids in meat

Freeze-dried and ground SM samples were used to analyze medium- and long-chain fatty acid contents. Firstly, lipids were extracted from SM samples by the chloroacetyl-methanol (1:10, v/v) procedure. Hexanes were added for methylation at 80°C water incubation for 4 h. Then, the gas chromatography was utilized to detect the profile of free fatty acids in samples by targeted metabolomics according to the description by Tang et al. (2020).

### Quantification of short-chain fatty acids

Approximately 1 g of cecal chyme was collected and immersed in 10 mL of ddH_2_O in 15-mL screw-capped vials, after which it was shaken for 30 min and set aside at 4°C overnight. For the analysis of SCFA concentration, the mixture was centrifuged at 10000 rpm for 10 min. The concentration was determined using gas chromatography according to Tang et al. (2021b).

### Microbial 16S rRNA gene sequencing analysis

Each sample contained approximately 0.5–1 g of cecal chyme, from which the manufacturer’s instructions of the E.Z.N.A.^®^ soil DNA Kit (D5625-02, Omega Bio-Tek Inc., Norcross, GA, United States) were followed to extract microbial community genomic DNA. A 1% agarose gel electrophoresis and NanoDrop2000 spectrophotometer (Thermo Fisher Scientific, Waltham, MA, United States) were used separately to determine the purity and DNA concentration. These primers [338F (5’-ACTCCTACGGGAGGCAGCAG-3′) and 806R (5’-GGACTACHVGGGTWTCTAAT-3′)] were used to amplify the V3-V4 regions of the 16S rRNA gene of bacteria. As described by Tang et al. (2021a), the reaction system, measuring amplified fragments, and purification were conducted according to their methods.

We obtained microbial sequence data from Majorbio Bio-Pharm Technology Co. Ltd. (Shanghai, China). Sequences were analyzed and classified into operational taxonomic units (**OTUs**; 97% identity). Additionally, QIIME (Version 174 1.7.0) generated alpha-diversity coverage based on the Ace, Chao and Sobs index within each sample, and an unweighted UniFrac distance based on Bray-Curtis distance was computed and PCoA was used to visualize beta-diversity. The significant difference between treatments at the phylum and genus level was tested by the Kruskal-Wallis H test (nonparametric test) with corrected *p*-value (FDR) < 0.05. Unless otherwise noted, the microbial data were analyzed on the Majorbio I-Sanger Cloud Platform.^1^

### Statistical analysis

One-way ANOVA analysis of the data on CW, serum metabolites, meat quality index, free fatty acids in meat, SCFAs in cecal chyme and microbial alpha-diversity (including Ace, Chao and Sobs index) was used to test for differences among 3 groups using the SPSS software (version 23.0, IBM, Armonk, NY, United States). The least significant difference (LSD) as a post-hoc multiple comparison method was used to compare results between every 2 of 3 distinct groups. The correlation matrix between cecal microbes and free fatty acids or SCFAs was generated using Spearman’s correlation coefficient on the Majorbio I-Sanger Cloud Platform. All the above data were drawn using GraphPad 7.0. Lastly, the results were presented as means ± SE with *p* < 0.05 being regarded as statistically significant.

## Results

### Carcass weight and serum metabolites

**Figure 1:**
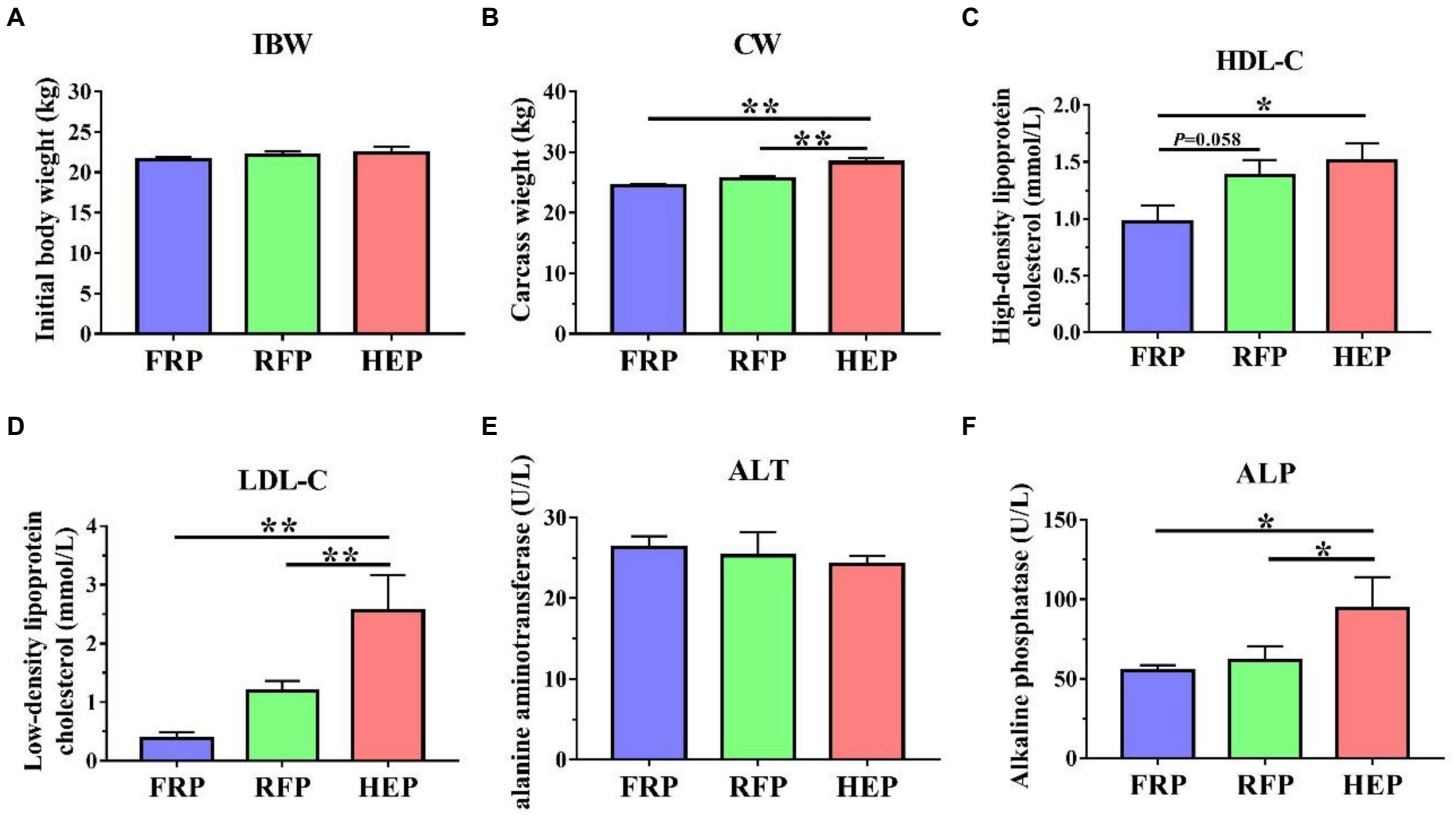
Effects of different feeding patterns on the CW, and HDL-C, LDL-C, ALT ALP in serum of Tibetan pigs. **(A)** The initial body weight (IBW) of Tibetan pigs. **(B)** The carcass weight (CW) of Tibetan pigs. **(C)** The content of high-density lipoprotein cholesterol (HDL-C) in serum of Tibetan pigs. **(D)** The content of low-density lipoprotein cholesterol (LDL-C) in serum of Tibetan pigs. **(E)** The activity of alanine aminotransferase (ALT) in serum of Tibetan pigs. **(F)** The activity of alkaline phosphatase (ALP) in serum of Tibetan pigs. Data are expressed as mean ± SE (*n* = 6–7). * and ** indicates *p* < 0.05 and *p* < 0.01, respectively. FRP: free-ranging Tibetan pigs; RFP: feeding the regular Tibetan pig diets; HEP: feeding the high energy content diets.

The IBW and CW, as well as HDL-C, LDL-C, ALT and ALP in serum were shown in Figure 1. The CW and LDL-C of HEP pigs were significantly higher than those of the FRP or RFP pigs (*p* < 0.01), whereas no significant changes in CW and LDL-C were found between FRP and RFP pigs (*p* > 0.05; Figures 1B,D). The HDL-C of HEP pigs was significantly improved compared with the FRP pigs (*p* < 0.05), and the HDL-C of RFP pigs had an increased trend compared with the FRP pigs (*p* = 0.058; Figure 1C). The ALP of HEP pigs was significantly higher compared with the FRP or RFP pigs (*p* < 0.05), whereas no significant change in ALP was found between FRP and RFP pigs (*p* > 0.05; Figure 1F). In addition, no significant difference was observed in ALT among three group pigs (*p* > 0.05; Figure 1E).

### Alterations in meat quality

The meat color, pH value, and shear force of SM were shown in Table 2. The L* (45 min), L* (24 h) and shear force of SM in FRP pigs were significantly higher than that in RFP and HEP pigs (*p* < 0.001), whereas no significant changes in L* (45 min), L* (24 h) and shear force were found between HEP and RFP pigs (*p* > 0.05). The a* (45 min) and a* (24 h) of RFP pigs were significantly lower compared with FRP pigs (*p* < 0.05). The b* (45 min) and pH_45min_ of HEP pigs were the highest, being significantly more elevated than those of RFP and FRP pigs (*p* < 0.05). However, the b* (24 h) and pH_24h_ of HEP pigs were the lowest and were significantly lower than that of the FRP pigs (*p* < 0.01).

**Table 2:** The meat quality of semitendinosus muscle.

Items		FRP	RFP	HEP	*p* value
Meat color	L* (45 min)	44.5610 ± 0.85633^a^	36.9057 ± 1.52944^b^	38.4371 ± 0.56005^b^	<0.001
	L* (24 h)	46.0100 ± 0.56874^a^	40.4414 ± 0.81403^b^	39.7986 ± 0.86017^b^	<0.001
	a* (45 min)	24.8580 ± 0.70553^a^	22.5186 ± 0.44134^b^	22.9286 ± 0.46605^ab^	0.022
	a* (24 h)	21.3890 ± 0.53471^a^	19.5286 ± 0.43414^b^	19.9100 ± 0.57722^ab^	0.042
	b* (45 min)	2.0830 ± 0.07926^b^	3.0314 ± 0.24751^b^	4.2529 ± 0.59320^a^	<0.001
	b* (24 h)	7.5190 ± 0.50205^a^	5.7529 ± 0.85541^ab^	4.2486 ± 0.67026^b^	0.006
pH values	pH_45min_	6.3522 ± 0.04099^b^	6.3457 ± 0.07211^b^	6.5614 ± 0.03508^a^	0.012
	pH_24h_	5.6833 ± 0.06254^a^	5.6071 ± 0.06672^a^	5.3829 ± 0.03797^b^	0.005
Shear force (N)		56.6240 ± 1.78240^a^	46.6219 ± 2.29898^b^	42.2857 ± 2.85427^b^	<0.001

Values in the same row with different superscripts (a, b) are significantly different (*p* < 0.05) (*n* = 6–7). L* (45 min) or L* (24 h): the lightness of meat color after slaughter 45 min or 24 h; a* (45 min) or a* (24 h): the redness of meat color after slaughter 45 min or 24 h; b* (45 min) or b* (24 h): the yellowness of meat color after slaughter 45 min or 24 h; pH_45min_ or pH_24h_: the pH value of meat after slaughter 45 min or 24 h. FRP: free-ranging Tibetan pigs; RFP: feeding the regular Tibetan pig diets; HEP: feeding the high energy content diets.

### Concentrations of free fatty acids in meat

**Figure 2:**
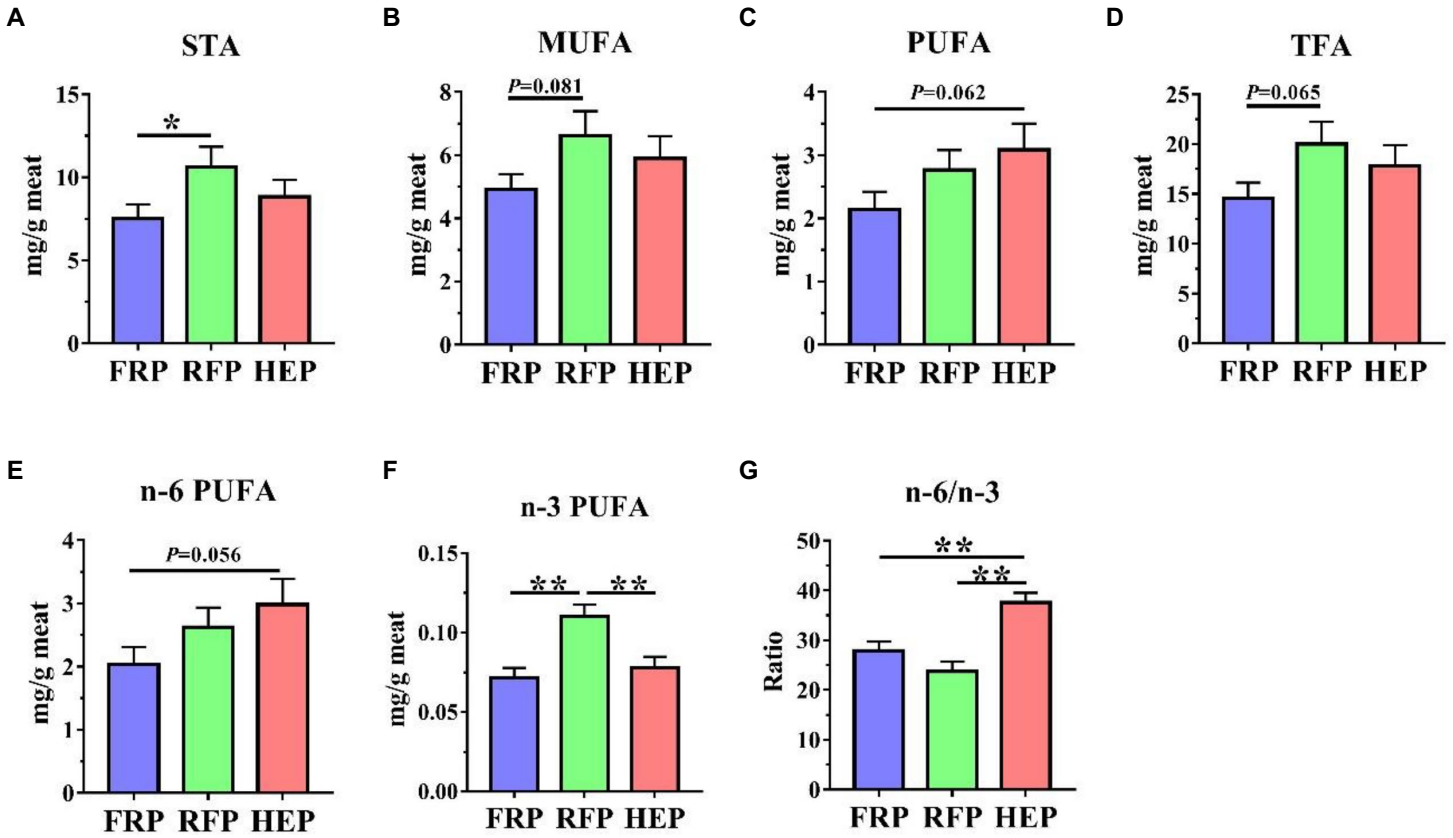
Effects of different feeding patterns on concentrations of fatty acids in semitendinosus muscle (SM) of Tibetan pigs. The concentrations of saturated fatty acid (STA, **A**), monounsaturated fatty acid (MUFA, **B**), polyunsaturated fatty acid (PUFA, **C**), total fatty acid (TFA, **D**), n-6 PUFA **(E)**, n-3 PUFA **(F)** and n-6/n-3 PUFA ratio **(G)** in SM of Tibetan pigs, respectively. Data are expressed as mean ± SE (*n* = 6–7). * and ** indicates *p*<0.05 and *p*<0.01, respectively. FRP: free-ranging Tibetan pigs; RFP: feeding the regular Tibetan pig diets; HEP: feeding the high energy content diets.

**Figure 3:**
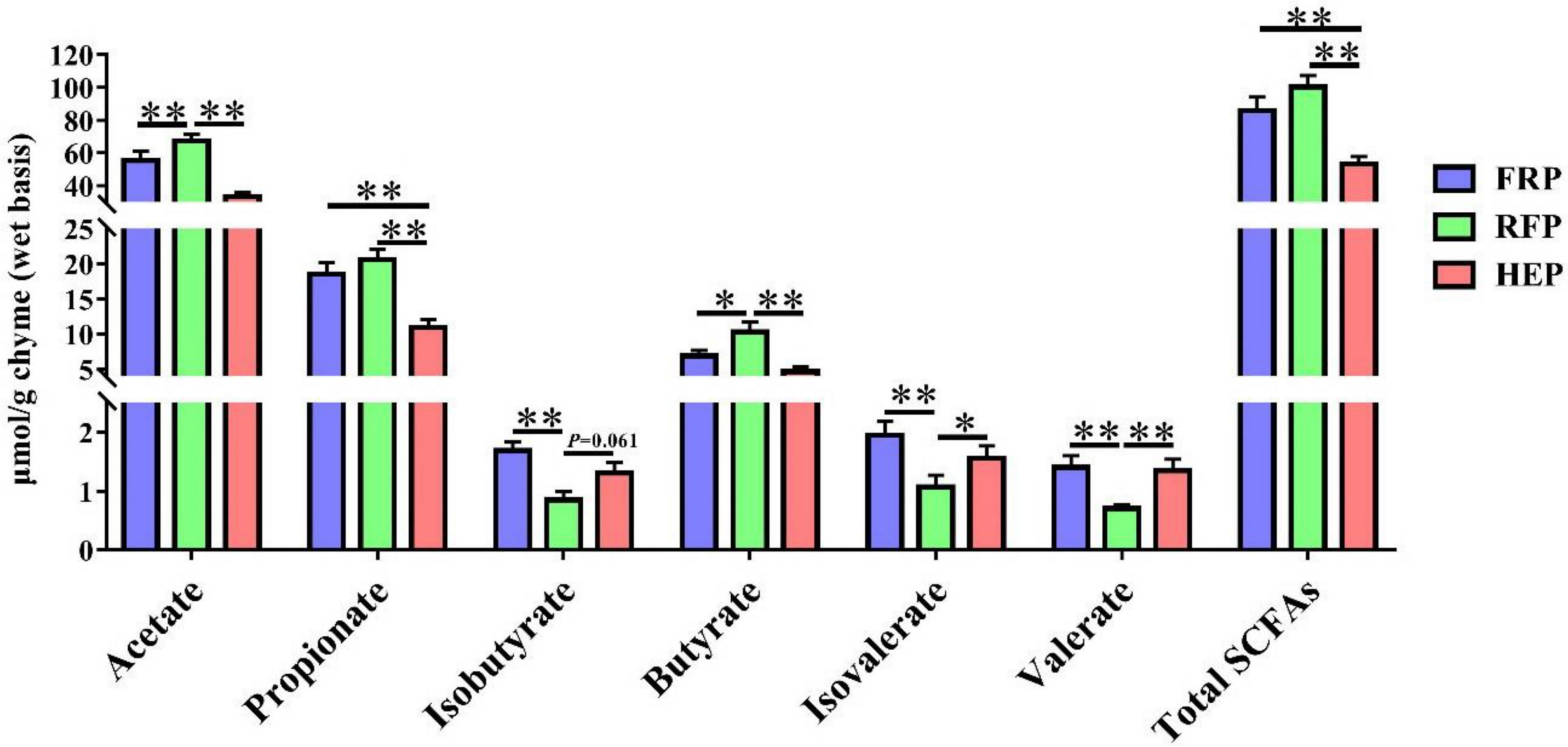
Effects of different feeding patterns on concentrations of short-chain fatty acid (SCFA), including acetate acid, propionic acid, isobutyric acid, butyric acid, isovaleric acid, valeric acid and total SCFAs in cecal chyme of Tibetan pigs. Data are expressed as mean ± SE (*n* = 6–7). * and ** indicates *p* < 0.05 and *p* < 0.01, respectively. FRP: free-ranging Tibetan pigs; RFP: feeding the regular Tibetan pig diets; HEP: feeding the high energy content diets.

**Figure 4:**
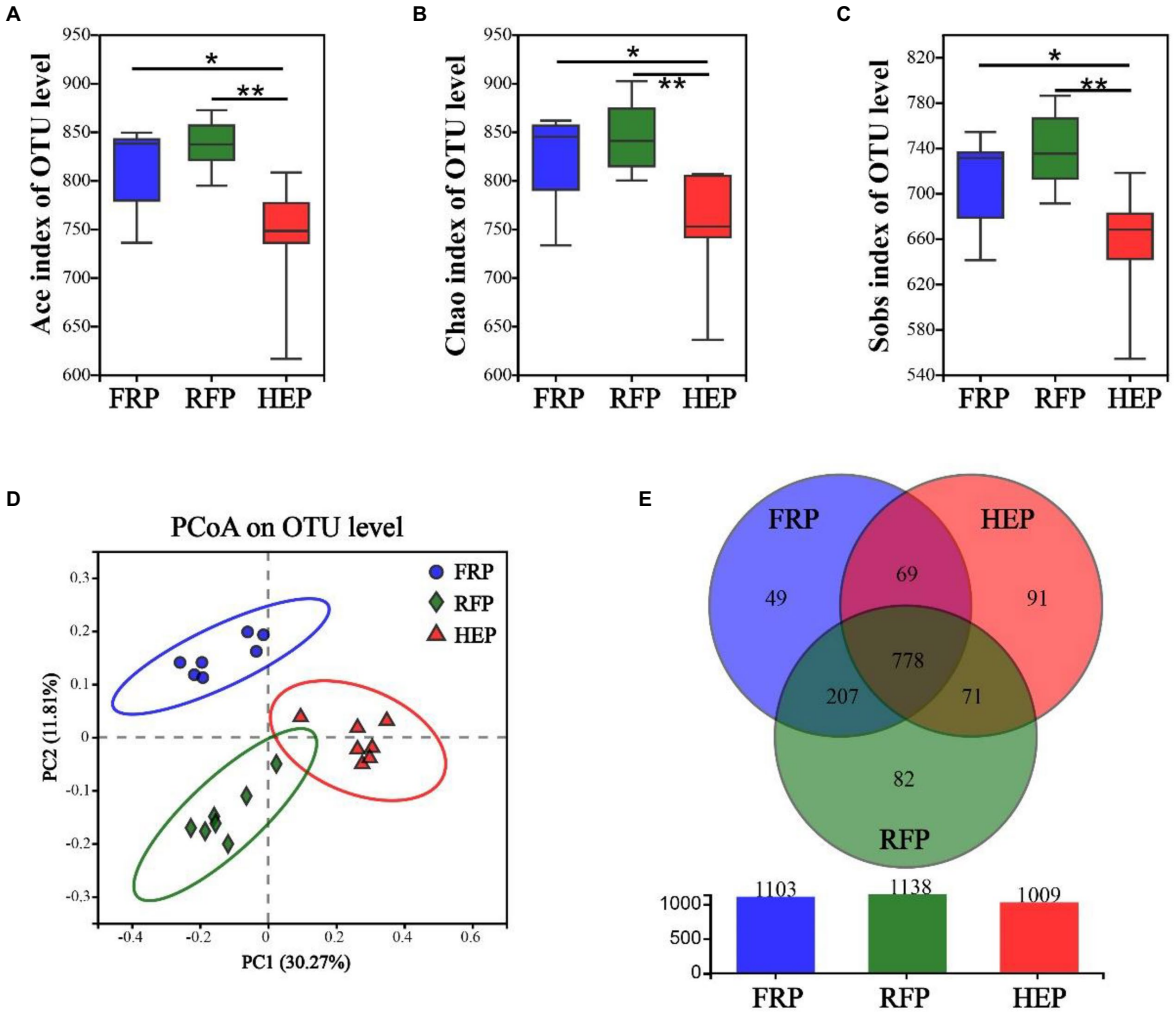
Effects of different feeding patterns on gut microbiota diversity of Tibetan pigs. The Ace **(A)**, Chao **(B)** and Sobs index **(C)** of microbiota in cecal chyme. Data are expressed as minimum to maximum (*n* = 7). * and ** indicates *p*<0.05 and *p*<0.01, respectively. **(D)** PCoA analysis of microbiota in cecal chyme at the OTU level based on the Bray-Curtis distance. **(E)** The Venn figure of microbiota in cecal chyme. FRP: free-ranging Tibetan pigs; RFP: feeding the regular Tibetan pig diets; HEP: feeding the high energy content diets.

**Figure 5:**
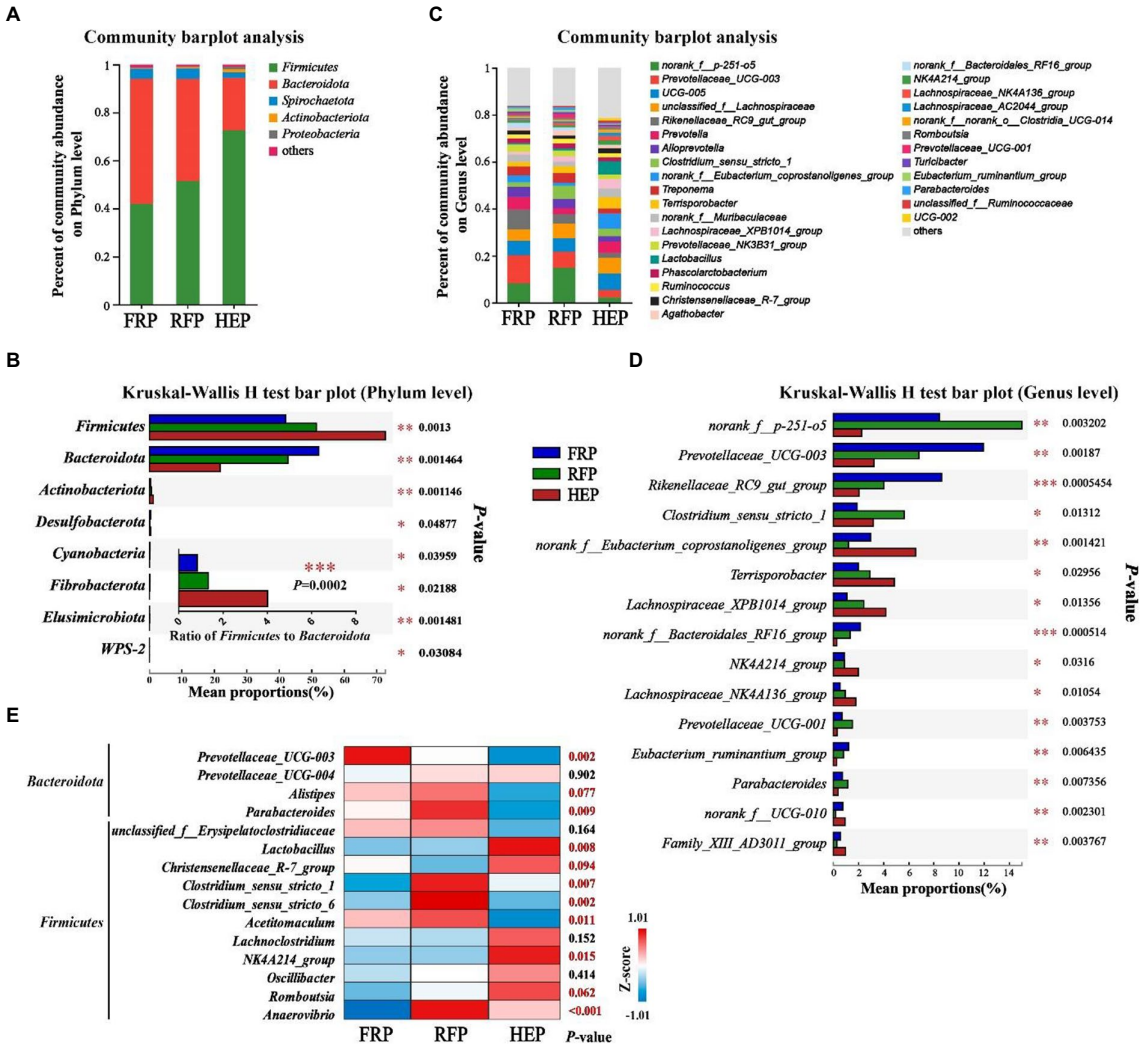
Effects of different feeding patterns on gut microbiota community composition in the cecal chyme of Tibetan pigs. The relative abundances of microbiota in cecal chyme at the phylum **(A)** or genus level **(C)**. Analysis of variance in the gut microbiota with significant differences at the phylum **(B)** and genus level (the top 15 genera, **D**). **(E)** heat map of the different enrichment of bacteria related to meat quality among three groups. Data are expressed as mean value (*n* = 7). *, ** and *** indicates *p*<0.05, *p*<0.01 and *p*<0.001, respectively. FRP: free-ranging Tibetan pigs; RFP: feeding the regular Tibetan pig diets; HEP: feeding the high energy content diets.

**Figure 6:**
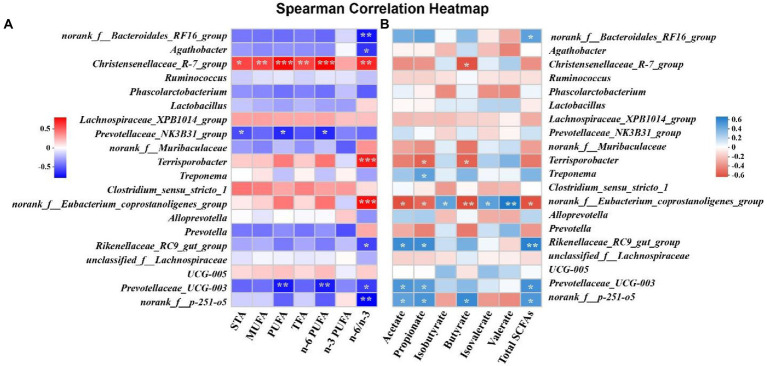
The correlation matrix of cecal genera, free fatty acids and short-chain fatty acids (SCFAs). **(A)** The correlation between cecal genera and free fatty acids in semitendinosus muscle. **(B)** The correlation between cecal genera and SCFAs in cecal chyme. *, ** and *** indicates *p*<0.05, *p*<0.01 and *p*<0.001, respectively. STA, saturated fatty acid; MUFA, monounsaturated fatty acid; PUFA, polyunsaturated fatty acid; TFA, total fatty acid.

The concentrations of free fatty acids in SM were shown in Figure 2. The concentrations of saturated fatty acid (**STA**, *p* < 0.05; Figure 2A), monounsaturated fatty acid (**MUFA**, *p* = 0.081; Figure 2B), total fatty acid (**TFA**, *p* = 0.065; Figure 2D) and n-3 polyunsaturated fatty acid (**PUFA**, *p* < 0.01; Figure 2F) in SM of RFP pigs were higher compared with FRP pigs, and the n-3 PUFA in RFP pigs was still higher than that in HEP pigs (*p* < 0.01; Figure 2F). The concentrations of PUFA (*p* = 0.062, Figure 2C) and n-6 PUFA (*p* = 0.056, Figure 2E) in HEP pigs had an increased trend compared with the FRP pigs. In addition, the ratio of n-6/n-3 PUFA in HEP pigs was significantly higher compared with the FRP and RFP pigs (*p* < 0.01), whereas no significant change in the ratio was found between FRP and RFP pigs (*p* > 0.05; Figure 2G).

As data shown in Table 3, the concentrations of C6:0, C20:2, C20:5 n3 and C22:0 of SM in FRP pigs were higher than that in HEP pigs (*p* < 0.05). The concentrations of C12:0, C14:0, C16:0, C17:0, C17:1, C18:0, C18:3 n3, C20:0, C20:1 n9, and C20:3 n3 of SM in RFP pigs were significantly higher compared with the FRP pigs (*p* < 0.05), whereas no significant change in concentrations of C12:0, C14:0, C16:0, C17:0, C17:1, C18:3 n3, and C20:1 n9 were found between HEP and RFP pigs (*p* > 0.05). The concentrations of C14:1 and C18:2,cis(n6) of SM in FRP pigs were lower compared with the RFP and HEP pigs, and significantly lower than that in the HEP pigs (*p* < 0.05). In addition, the concentration of C20:4 n6 of RFP pigs was the highest among three group pigs, and significantly higher than that in the HEP pigs (*p* < 0.05).

**Table 3:** The concentrations of free fatty acids in semitendinosus muscle.

Items	FRP	RFP	HEP	*p*-value
C6:0	0.0071 ± 0.00070^a^	0.0055 ± 0.00010^ab^	0.0045 ± 0.00030^b^	0.011
C8:0	0.0029 ± 0.00015	0.0032 ± 0.00020	0.0031 ± 0.00018	0.557
C10:0	0.0133 ± 0.00113	0.0160 ± 0.00163	0.0148 ± 0.00166	0.405
C12:0	0.0086 ± 0.00106^b^	0.0141 ± 0.00173^a^	0.0150 ± 0.00202^a^	0.013
C14:0	0.1224 ± 0.01747^b^	0.2197 ± 0.02990^a^	0.2199 ± 0.03506^a^	0.017
C14:1	0.0041 ± 0.00024^b^	0.0052 ± 0.00038^ab^	0.0066 ± 0.00094^a^	0.01
C16:0	4.9156 ± 0.54760^b^	7.6975 ± 0.94717^a^	6.6303 ± 0.83387^ab^	0.042
C16:1	0.3261 ± 0.03787	0.4642 ± 0.05147	0.5347 ± 0.09375	0.055
C17:0	0.0363 ± 0.00603^b^	0.0636 ± 0.01085^a^	0.0397 ± 0.00570^ab^	0.045
C17:1	0.0271 ± 0.00416^b^	0.0467 ± 0.00718^a^	0.0329 ± 0.00465^ab^	0.047
C18:0	1.5339 ± 0.15239^b^	2.1517 ± 0.24149^a^	1.5179 ± 0.13221^b^	0.042
C18:1,trans(n9)	0.0419 ± 0.11920	0.0310 ± 0.01596	0.0056 ± 0.00555	0.128
C18:1,cis(n9)	3.9440 ± 0.43034	5.8624 ± 0.69633	5.1484 ± 0.60341	0.062
C18:2,trans(n6)	0.2908 ± 0.08110	0.6042 ± 0.08765	0.5198 ± 0.10835	0.056
C18:2,cis(n6), LA	1.5852 ± 0.15389^b^	1.9638 ± 0.23539^ab^	2.4176 ± 0.30595^a^	0.047
C18:3 n6	0.0092 ± 0.00068	0.0075 ± 0.00057	0.0100 ± 0.00171	0.269
C18:3 n3, ALA	0.0207 ± 0.00257^b^	0.0357 ± 0.00374^a^	0.0277 ± 0.00379^ab^	0.014
C20:0	0.0488 ± 0.00784^b^	0.1306 ± 0.02530^a^	0.0802 ± 0.12700^b^	0.004
C20:1 n9	0.1001 ± 0.00947^b^	0.1833 ± 0.02523^a^	0.1453 ± 0.01975^ab^	0.009
C20:2	0.0196 ± 0.00238^a^	0.0152 ± 0.00141^ab^	0.0090 ± 0.00171^b^	0.007
C20:3 n6	0.0466 ± 0.00487	0.0356 ± 0.00348	0.0363 ± 0.00498	0.18
C20:4 n6, ARA	0.0061 ± 0.00131^ab^	0.0137 ± 0.00538^a^	0.0011 ± 0.00059^b^	0.029
C20:3 n3	0.0273 ± 0.00153^b^	0.0463 ± 0.00507^a^	0.0308 ± 0.00153^b^	<0.001
C20:5 n3, EPA	0.0158 ± 0.00092^a^	0.0154 ± 0.00030^a^	0.0128 ± 0.00066^b^	0.035
C22:0	0.4347 ± 0.05281^a^	0.2681 ± 0.01530^b^	0.2646 ± 0.03119^b^	0.013
C22:1	0.0007 ± 0.00052	<0.00001	<0.00001	0.296
C22:2	0.0160 ± 0.00144	0.0142 ± 0.00060	0.0120 ± 0.00142	0.129
C22:6 n3, DHA	0.0111 ± 0.00209	0.0128 ± 0.00266	0.0067 ± 0.00204	0.213
C24:0	0.0478 ± 0.00379	0.0419 ± 0.00202	0.0385 ± 0.00428	0.2
C24:1	0.0253 ± 0.00242	0.0242 ± 0.00123	0.0211 ± 0.00244	0.421

Values in the same row with different superscripts (a, b) are significantly different (*P* < 0.05) (*n* = 6–7). The results were expressed as free fatty acid (mg) per 1 g of freeze-dried meat. C6:0: caproic acid; C8:0: octanoic acid; C10:0: decanoic acid; C12:0: lauric acid; C14:0: myristic acid; C14:1: tetradecenoic acid; C16:0: palmitic acid; C16:1: palmitoleic acid; C17:0: margaric acid; C17:1: margaroleic acid; C18:0: stearic acid; C18:1,trans(n9): elaidic acid; C18:1,cis(n9): oleic acid; C18:2,trans(n6): trans-linoleic acid n6; C18:2,cis(n6): linoleic acid n6; C18:3 n6: gamma-linolenic acid; C18:3 n3: alpha-linolenic acid; C20:0: arachidic acid; C20:1 n9: eicosenoic acid n9; C20:2: eicosadienoic acid; C20:3 n6: eicosatrienoic acid n6; C20:4 n6: arachidonic acid n6; C20:3 n3: eicosatrienoic acid n3; C20:5 n3: eicosapentaenoic acid n3; C22:0: behenic acid; C22:1: cetoleic acid; C22:2: docosadienoic acid; C22:6 n3: docosahexaenoic acid n3; C24:0: lignoceric acid; C24:1: nervonic acid. FRP: free-ranging Tibetan pigs; RFP: feeding the regular Tibetan pig diets; HEP: feeding the high energy content diets.

### Concentrations of short-chain fatty acids in cecal chyme

The concentrations of SCFAs in cecal chyme were shown in Figure 3. The levels of acetic acid and butyric acid in RFP pigs were higher than that in FRP and HEP pigs (*p* < 0.05), whereas no significant changes of that were found between HEP and FRP pigs (*p* > 0.05). The levels of propionic acid and total SCFAs in cecal chyme of FRP or RFP pigs were higher compared with HEP pigs (*p* < 0.01). The concentrations of isobutyrate, isovalerate and valerate in RFP pigs were lower compared with FRP or HEP pigs (*p* < 0.05).

### Variations in cecal microbes

The fresh cecal chyme was obtained from FRP, RFP and HEP pigs, and 16 s rRNA gene sequencing analysis was performed. The Ace (Figure 4A), Chao (Figure 4B) and Sobs index (Figure 4C) of cecal chyme microbes at OTU level in HEP pigs were significantly lower than that in FRP pigs (*p* < 0.05), and extremely significantly lower than that in RFP pigs (*p* < 0.01). The PCoA analysis based on Bray-Curtis distance revealed that beta-diversity shifted due to distinct feeding patterns and notable differences were observed in the cecal chyme at the OTU level (Figure 4D). There were 1103 (208), 1138 (232) and 1009 (214) OTUs (genera) obtained from FRP, RFP and HEP pigs, respectively, of which 778 (181) were common OTUs (genera) among the three different feeding patterns (Figure 4E).

Microbial community composition at the phylum and genus level of the three feeding patterns was presented in Figure 5. The cecal chyme samples comprised five major phyla including *Firmicutes*, *Bacteroidota*, *Spirochaetota*, *Actinobacteria* and *Proteobacteria*, and the *Firmicutes* and *Bacteroidetes* were the most predominant phyla in the cecal chyme of FRP, RFP and HEP pigs (Figure 5A). In addition, there were also significant differences in the abundance of *Firmicutes* and *Bacteroidota*, as well as the ratio of *Firmicutes* to *Bacteroidota* among three groups (*p* < 0.01; Figure 5B). At the genus level, the top three most abundant genera in different feeding patterns, in turn, were *norank_f_p-251-o5, Prevotellaceae_UCG-003* and *Rikenellaceae_RC9_gut_group* (Figures 5C,D). The *Prevotellaceae_UCG-003* was extremely significantly enriched in the FRP pigs (*p* < 0.01); the *Parabacteroides, Clostridium_sensu_stricto_1, Clostridium_sensu_stricto_6,* and *Anaerovibrio* were extremely significantly enriched in the RFP pigs (*p* < 0.01); and *Lactobacillus* was extremely significantly enriched in the HEP pigs (*p* < 0.01; Figure 5E).

### Correlation between microbiota and free fatty acids in meat or SCFAs in cecal chyme

Spearman’s correlation analysis between microbiota and free fatty acids or SCFAs were shown in Figure 6. The relative abundance of *Christensenellaceae_R-7_group* was positively associated with the levels of STA, MUFA, PUFA, TFA, n-6 PUFA and n-6/n-3 ratio in SM, except for n-3 PUFA (*p* < 0.05, Figure 6A). The relative abundance of *Terrisporobacter* and *norank_f_Eubacterium_coprostanoligenes_group* were positively associated with the ratio of n-6/n-3 in SM (*p* < 0.05), whereas the ratio of n-6/n-3 in SM was negatively correlated with the relative abundance of *norank_f_bacteroidales_RF16_group* and *norank_f_p-251-o5* (*p* < 0.05; Figure 6A). In addition, we found that the concentrations of PUFA and n-6 PUFA in SM were negatively correlated with the relative abundance of *Prevotallaceae_UCG-003* (*p* < 0.05, Figure 6A). The concentrations of valerate and total SCFAs were positively associated with the relative abundance of *Rikenellaceae_RC9_gut_group* and *norank_f_Eubacterium_coprostanoligenes_group*, respectively (*p* < 0.05, Figure 6B). However, the concentration of butyrate was negatively correlated with the relative abundance of *norank_f_Eubacterium_coprostanoligenes_group* (*p* < 0.01, Figure 6B).

## Discussion

Tibetan pig breeding, not only the focus of the development of agriculture and animal husbandry with local characteristics, but also a crucial industry for people in Tibetan areas to get rid of poverty and become rich, is a vital part of traditional animal husbandry in Tibet. As is known to all, the traditional Tibetan pig breeding method is mainly grazing with a long breeding cycle, which cannot be scientifically managed to maximize the utilization of Tibetan pig resources (Wang et al., 2013; Yang et al., 2021). House feeding can provide full mixed diet for Tibetan pigs without foraging and less exercise, which is conducive to muscle and fat deposition (Zhang et al., 2019). In this study, the carcass weight of Tibetan pigs in HEP group was significantly higher than those in RFP and FRP groups, indicating that the house feeding pattern with whole mixed diet was beneficial to promote the growth of Tibetan pigs and produce more meat, which was consistent with the results of Zhang et al. (2019).

The serum lipid-related metabolites may reflect the overall metabolic state to a certain extent (Tang et al., 2021b). The HDL-C, mainly synthesized in the liver, is an anti-atherogenic lipoprotein that transports cholesterol from extrahepatic tissues to the liver for metabolism and is excreted from the body by bile (Rigotti et al., 2003). By contrast, the LDL-C is a lipoprotein particle that carries cholesterol into peripheral tissue cells (van der Wulp et al., 2013). In this study, it was found that the levels of HDL-C and LDL-C in Tibetan pig varies due to the different energy intake of feeding patterns. The contents of LDL-C and HDL-C in serum of HEP group pigs were the highest, indicating that cholesterol turnover and metabolism in liver were increased. Similar results were also found in broilers with increased dietary energy levels causing elevated serum HDL and LDL levels (Hu et al., 2021). In addition, it was found in this study that the serum ALP level of Tibetan pigs under different feeding patterns was within the normal range (40–160 U/L), but the ALP level in Tibetan pigs fed high energy content diet was significantly higher than that in Tibetan pigs fed regular diet or free-ranging, meaning that the liver metabolic process was significantly enhanced (Siller and Whyte, 2018).

Tibetan pork, as an ecological, green and high-quality pork product, has attracted public attention. Meat quality is a crucial economic trait, which directly affects consumers’ preference for pork, and different meat quality characteristics are applied to different meat processing methods (Modzelewska-Kapitula et al., 2012; Wang et al., 2022a). For example, tender meat is suitable for grilling, while chewy meat is better for braising. This study showed that differences in energy intake levels caused by different feeding patterns did result in alterations in meat quality (including tenderness, pH value, meat color, medium and long-chain fatty acid content). We observed that the shear force of Tibetan pork in the grazing group was significantly higher than those of the other two groups, and the meat color was brighter and redder (L* and a* values). The type and composition of muscle fibers were closely related to meat quality, and the type I myofiber was more enriched in the muscle of grazing Tibetan pigs due to frequent exercise and running (Jackson et al., 2015). The meat color depends on the content of myoglobin in the muscle (Bekhit and Faustman, 2005), and the free-range Tibetan pork was red and bright because of increased type I myofiber, oxidative myofiber, with high content of myoglobin (Ryu and Kim, 2005). The reason why Tibetan pork became dark red with the increase of exposure time under any feeding pattern was that oxymyoglobin (bright red) was oxidized to methemoglobin (brown) (Bekhit and Faustman, 2005). In addition, the researcher believed that the glycolytic myofiber (type IIb) has a faster maturation rate than the oxidative myofiber (type I), so the higher proportion of type I myofiber exhibited a higher shear force in muscle (Seideman, 1986), which provided a reasonable explanation for the alterations of pork tenderness under different feeding pattern in this experiment. Generally, meat with a pH_45min_ ≥ 6 measured after 45 min of slaughter is considered as high-quality meat, and meat with a pH_24h_ > 6 measured after 24 h is considered as dark firm dry meat (Yin, 2011). Hence, from the point of view of pH value, we found high quality of Tibetan pork in any feeding pattern, and the highest pH_24h_ was found in the free-ranging group. Due to the high proportion of type I myofiber with low glycogen content and weak glycolytic ability (Yin, 2011) in free-ranging Tibetan pigs, the lactic acid production was low, finally leading to higher pH_24h_ in muscle. Intriguingly, the pH value of Tibetan pigs in free-ranging group and regular diet feeding group at 45 min was lower than that of Tibetan pigs in high energy group, which might be closely related to dietary fiber content.

Additionally, fatty acids are essential for human health with various physiological functions (Calder, 2015). We observed that the total fatty acids and unsaturated fatty acids in meat of Tibetan pigs fed high energy diets and regular diets were significantly higher than those in free-ranging Tibetan pigs, indicating the crucial roles of energy intake level for the fatty acid content in meat (Wang et al., 2020). It is widely recognized that n-6 PUFAs have potentially negative effects, while n-3 PUFAs [especially eicosapentaenoic acid (**EPA**) and docosahexaenoic acid (**DHA**)] have significant positive effects on human health (Jeromson et al., 2015; Sears and Perry, 2015). Meanwhile, researchers have demonstrated that n-3 PUFA exhibited curative effects on bronchial asthma, neuropsychiatric disorders and cognitive brain function in children and can also prevent future cardiovascular events in adults (Ciccone et al., 2013). The fatty acid composition, especially n-3 PUFA, is modulated by altering dietary lipid intake and absorption levels (Poorghasemi et al., 2013). In this study, Tibetan pigs fed regular diets had more reasonable fatty acid composition with higher n-3 PUFAs and lower n-6/n-3 PUFA ratio than high energy intake Tibetan pigs. In addition to n-3 PUFAs, other fatty acids including C18:3n-3 (alpha-linolenic acid, **ALA**), C20:5n-3 (EPA) and C22:6n-3 (DHA) also have health benefits in preventing brain, retina and cardiovascular diseases (Howe et al., 2006). In this research, the higher content of EPA in free-ranging and regular diets group pork might be closely related to the alterations of gut microbiota caused by fiber intake (Chen et al., 2022). For other unsaturated fatty acids [linoleic acid (**LA**), ALA and arachidonic acid (**ARA**)] that are beneficial to human beings, different feeding patterns have their own advantages.

In recent years, the interaction between gut microbiota and muscle has been confirmed by more and more studies, and the importance of its microbiota-gut-muscle axis has been fully recognized (Ticinesi et al., 2019). Gut microbiota can affect skeletal muscle metabolism and muscle fiber phenotype (Lahiri et al., 2019; Nay et al., 2019), and several species of bacteria also have similar effects on regulating skeletal muscle metabolism (Ni et al., 2019; Scheiman et al., 2019). Hence, we analyzed the microbial composition in the cecum of Tibetan pigs, and the results exhibited that the microbial community was obviously divided into three different clusters under different feeding patterns, and the microbial diversity in the cecum of Tibetan pigs fed high energy diets was significantly reduced due to the lack of fiber in the diets (Pu et al., 2022). Besides, microbiota-derived SCFA was affected by dietary fiber intake (Pu et al., 2022), which was consistent with the results that the content of SCFAs (especially acetic acid, propionic acid, and butyric acid) was higher in the cecum of Tibetan pigs in free-ranging and regular diet groups with more fiber intake in this study. The results of Spearman correlation analysis suggested that the genera *Prevotellaceae UCG-003*, *norank_f_Eubacterium_coprostanoligenes_group*, *Rikenellaceae_RC9_gut_group* and *norank_f_p-251-o5* were significantly correlated with the production of SCFA in intestinal tract of Tibetan pigs. It has been reported that SCFA infusion from ileum inhibited the expression of fatty acid synthase and acetyl-CoA carboxylase in longissimus dorsi muscle and altered meat quality after antibiotic clearance of endogenous SCFA-produced microbiota in hindgut of pigs (Jiao et al., 2021). Feeding alfalfa and SCFA in goats significantly increased the content of C16:0 and C18:0 in meat (Wang et al., 2022b), which was highly similar to the results of this experiment. Simultaneously, increasing evidence confirmed that gut microbes (for example, *Parabacteroides*, *Unclassified Erysipelotrichaceae*, *Prevotellaceae UCG-001*, *Butyrivibrio*, *Alistipes*, *Phocaeicola*, *Acetitomaculun*, *Corynebacterium*, *Anaerovibrio*, *Lachnoclostridium_1*) affect lipid deposition and fatty acid content in skeletal muscle by altering lipid metabolism (Chen et al., 2022). A total of 11 genera related to lipid metabolism, including *Unclassified_f_Erysipelotrichaceae*, *Alistipes*, *Anaerovibrio*, *Acetitomaculun*, etc., were identified under different feeding patterns in this study. Among that, the high content of C18:1 cis-9 and C18:2n-6 trans in Tibetan pork with higher energy intake (REP and HEP group pigs) might be caused by the increased abundance of *Anaerovibrio* bacteria in intestinal tract (Wang et al., 2019). Besides, the high content of saturated fatty acids in Tibetan pork with higher energy intake was also closely related to the probiotic and antioxidant effects of *Lactobacillus* (*Lactobacillus johnsonii*) in intestinal tract (Wang et al., 2017). In this study, Spearman correlation analysis demonstrated that alterations in the content of medium or long-chain fatty acids in meat were affected by the genera *Prevotellaceae_NK3B31_group*, *Prevotellaceae UCG-003* and *Christensenellaceae_R-7_group*. In addition to the bacteria related to lipid metabolism, this study also found that the bacteria *Prevotella* and *Clostridium* related to meat quality (Fang et al., 2017) changed significantly in the cecum of Tibetan pigs under different feeding patterns.

## Conclusion

Taken together, our results demonstrated that high energy feeding pattern for house feeding improved carcass weight and enhanced feeding efficiency of Tibetan pigs, yet distinct feeding patterns affected meat quality of Tibetan pigs closely associated with altering gut microbiota, which could provide a reference for choosing specific feeding pattern based on various market orientation and demand to achieve precision breeding.

## Data availability statement

The datasets presented in this study can be found in online repositories. The names of the repository/repositories and accession number(s) can be found at: https://www.ncbi.nlm.nih.gov, PRJNA899682.

## Ethics statement

The animal study was reviewed and approved by the Institutional Animal Care and Use Committee of the Institute of Animal Sciences, Chinese Academy of Agricultural Sciences (Ethics Approval Code: IAS2021-241).

## Author contributions

YZ, CL, ST, and RZ designed the research. YZ, Cy, CL, GS, BS, and JD conducted the experiments. YZ and ST analyzed the data. YZ, RZ, Bw, and ST wrote the paper and revised the manuscript. YZ, TM, RZ, LC, and HZ provided the funding and supervision. All authors contributed to the article and approved the submitted version.

## Funding

This study was funded by the Tibet Science and Technology Projects (XZ-2019-NK-NS-003), the National Key Research and Development Program of China (2022YFD1600201), and Agricultural Science and Technology Innovation Program (CAAS-ZDRW202006-02, ASTIPIAS07).

## Conflict of interest

The authors declare that the research was conducted in the absence of any commercial or financial relationships that could be construed as a potential conflict of interest.

## Publisher’s note

All claims expressed in this article are solely those of the authors and do not necessarily represent those of their affiliated organizations, or those of the publisher, the editors and the reviewers. Any product that may be evaluated in this article, or claim that may be made by its manufacturer, is not guaranteed or endorsed by the publisher.

## Footnotes

^1^ https://cloud.majorbio.com
